# Supplementary material for: Effect of Using Personalized Estimates of Diabetes Risk During Primary Care Visits for People With Prediabetes
Source: Learn Health Syst. 2026 May 27;10(Suppl 1):e70087. doi: 10.1002/lrh2.70087 (PMC13240528; doi:10.1002/lrh2.70087)
Supplement: Supplementary file 1 — Figure S1: Diabetes risk prediction model parameters, retrieved from patient's record in Premier Medical Associates' Allscripts TouchWorks EHR, as displayed for review and editing before calculating model results (upon pressing button in lower right corner), at the time of the implementation study. This was one of several calculators implemented using the Galen eCalcs add‐in for TouchWorks (left column). Figure S2: Diabetes risk prediction model results, as displayed in Premier Medical Associates' Allscripts TouchWorks EHR at the time of the implementation study. [file LRH2-10-e70087-s001.pptx]

## Slide 1
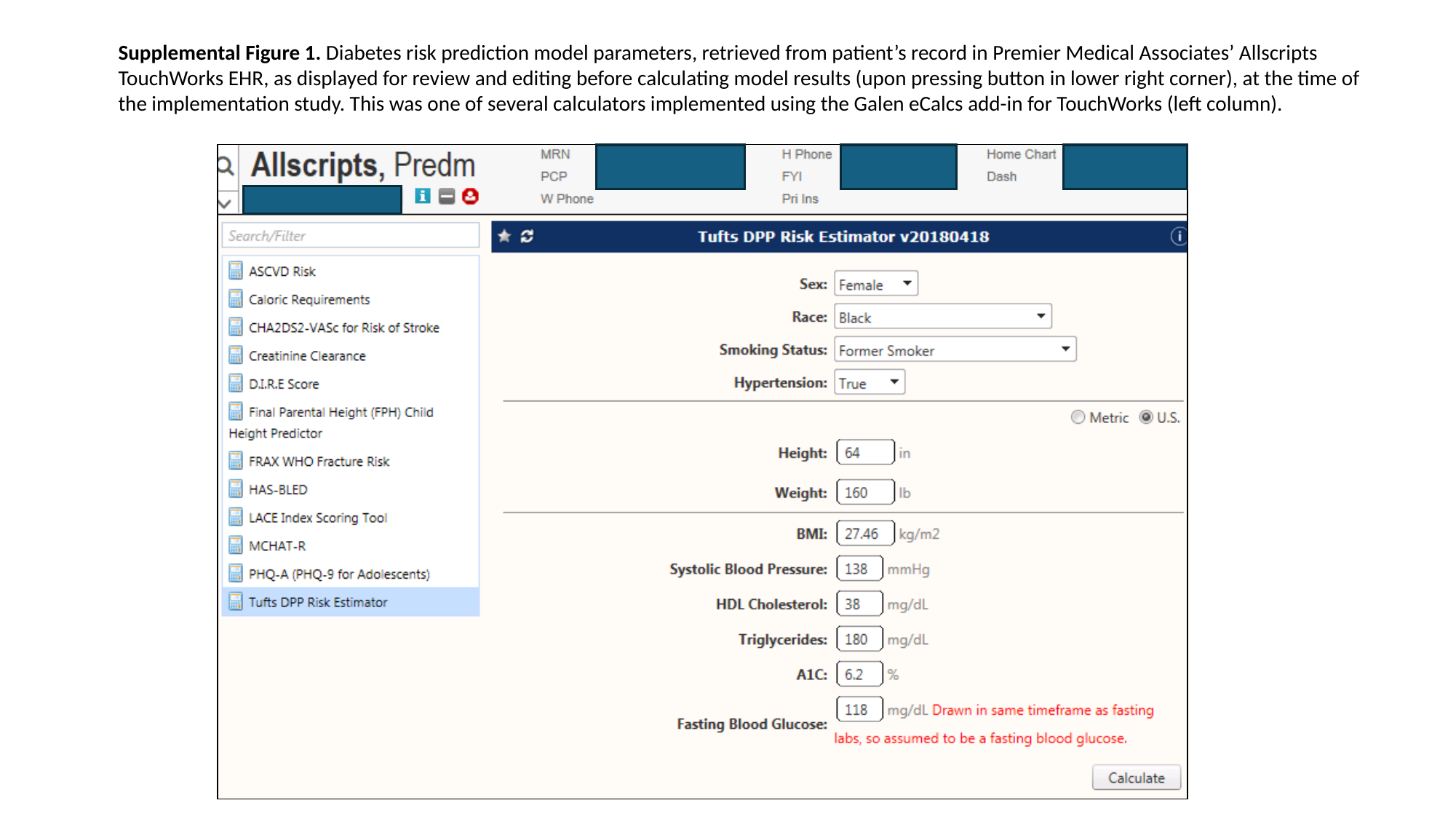

Supplemental Figure 1. Diabetes risk prediction model parameters, retrieved from patient’s record in Premier Medical Associates’ Allscripts TouchWorks EHR, as displayed for review and editing before calculating model results (upon pressing button in lower right corner), at the time of the implementation study. This was one of several calculators implemented using the Galen eCalcs add-in for TouchWorks (left column).

## Slide 2
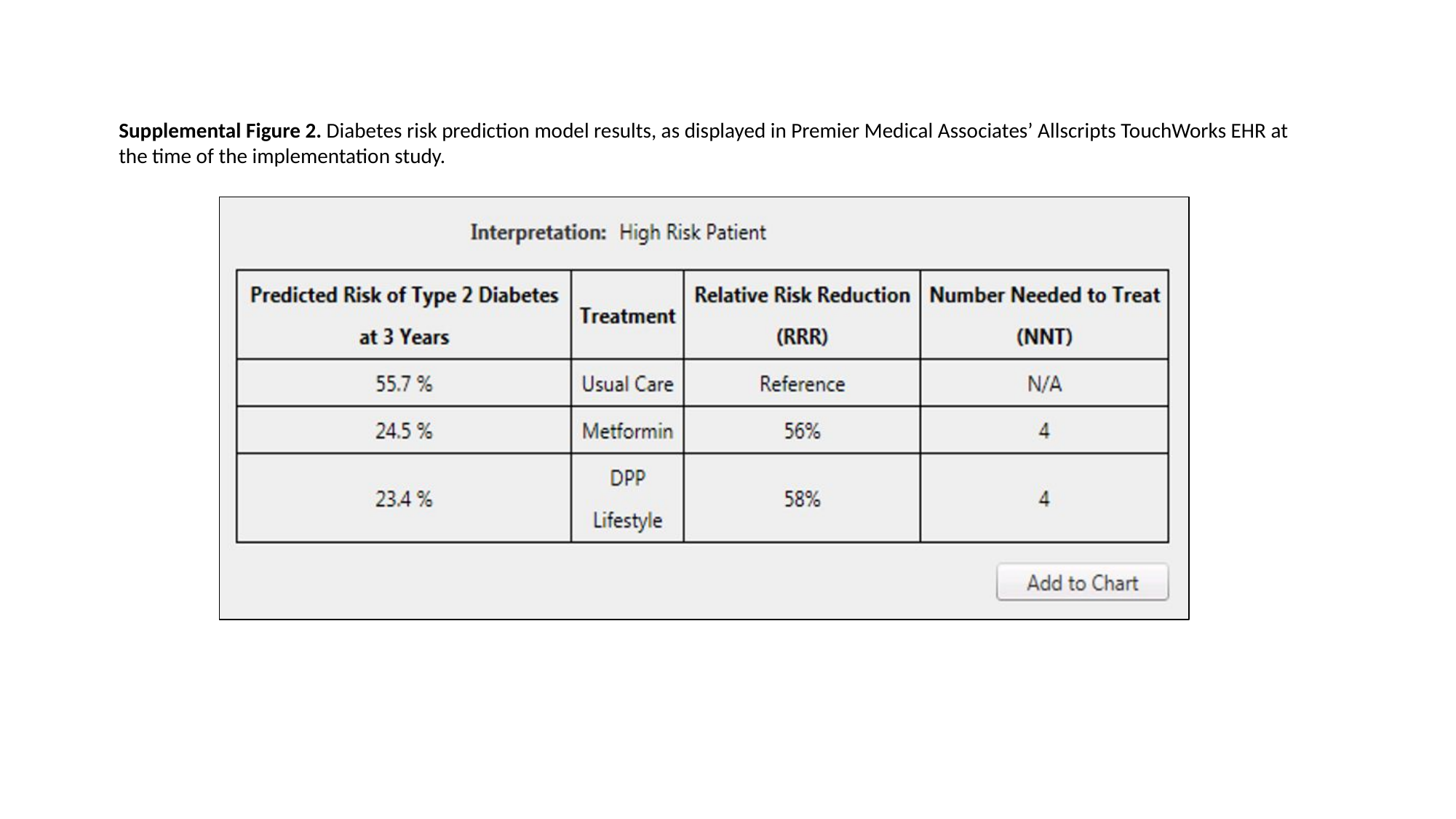

Supplemental Figure 2. Diabetes risk prediction model results, as displayed in Premier Medical Associates’ Allscripts TouchWorks EHR at the time of the implementation study.
