# Supplementary material for: Effect of Using Personalized Estimates of Diabetes Risk During Primary Care Visits for People With Prediabetes
Source: Learn Health Syst. 2026 May 27;10(Suppl 1):e70087. doi: 10.1002/lrh2.70087 (PMC13240528; doi:10.1002/lrh2.70087)

Supplemental File 2: Outcome Study Additional Tables and Figures

I. Definition of Progression to Diabetes

Progression to Diabetes based on claims data is defined as follows:

1. Type 2 – two or more distinct claims with E11.xx diagnosis in primary or any secondary field, or
2. Rx claim(s) in the post-period for a total of 60 days or more of a diabetes drug from **Table A** and no type 1 diagnosis code on any claim in the post-period and a type 2 diagnosis code on at least one post-period claim, or
3. Rx claim(s) in the post-period for a total of 60 days or more of a diabetes drug from **Table B**, and a type 2 diagnosis code on at least one post-period claim.

Table A

STC Code	STC Description
0178	Antihyperglycemic, insulin release stimulant type
0179	Antihyperglycemic, biguanide type
7768	Antihyperglycemic, alpha-glucosidase inhibitors
7769	Antihyperglycemic, thiazolidinedione (PPARG Agonist)
A716	Antihyperglycemic, amylin analog-type
B135	Antihyperglycemic, thiazolidinedione-sulfonylurea
B137	Antihyperglycemic, insulin-release stim. -Biguanide
B139	Antihyperglycemic, thiazolidinedione and biguanide
B789	Antihyperglycemic, DPP-4 inhibitors
C118	Antihyperglycemic, DPP-4 inhibitor-biguanide combs
D549	Antihyperglycemic, dopamine receptor agonists
E191	Antihyperglycemic, glucocorticoid receptor blocker
E874	Antihyperglycemic, DPP-4 inhibitor-thiazolidinedione combs
F555	Antihyperglycemic, SGLT2 inhibitor-biguanide combs
F883	Antihyperglycemic, SGLT2 and DPP-4 inhibitor comb

Table B

STC Code	STC Description
0179	Antihyperglycemic, biguanide type
A771	Antihyperglycemic, incretin mimetic (GLP-1 Receptor agonist)
E948	Antihyperglycemic-sodium/glucose cotransporter (SGLT2) inhibitor
0277	Bromocriptine mesylate (Antiparkinsonism drug, other)

II. Sample Selection for Outcome Analysis

A. Commercially insured patients

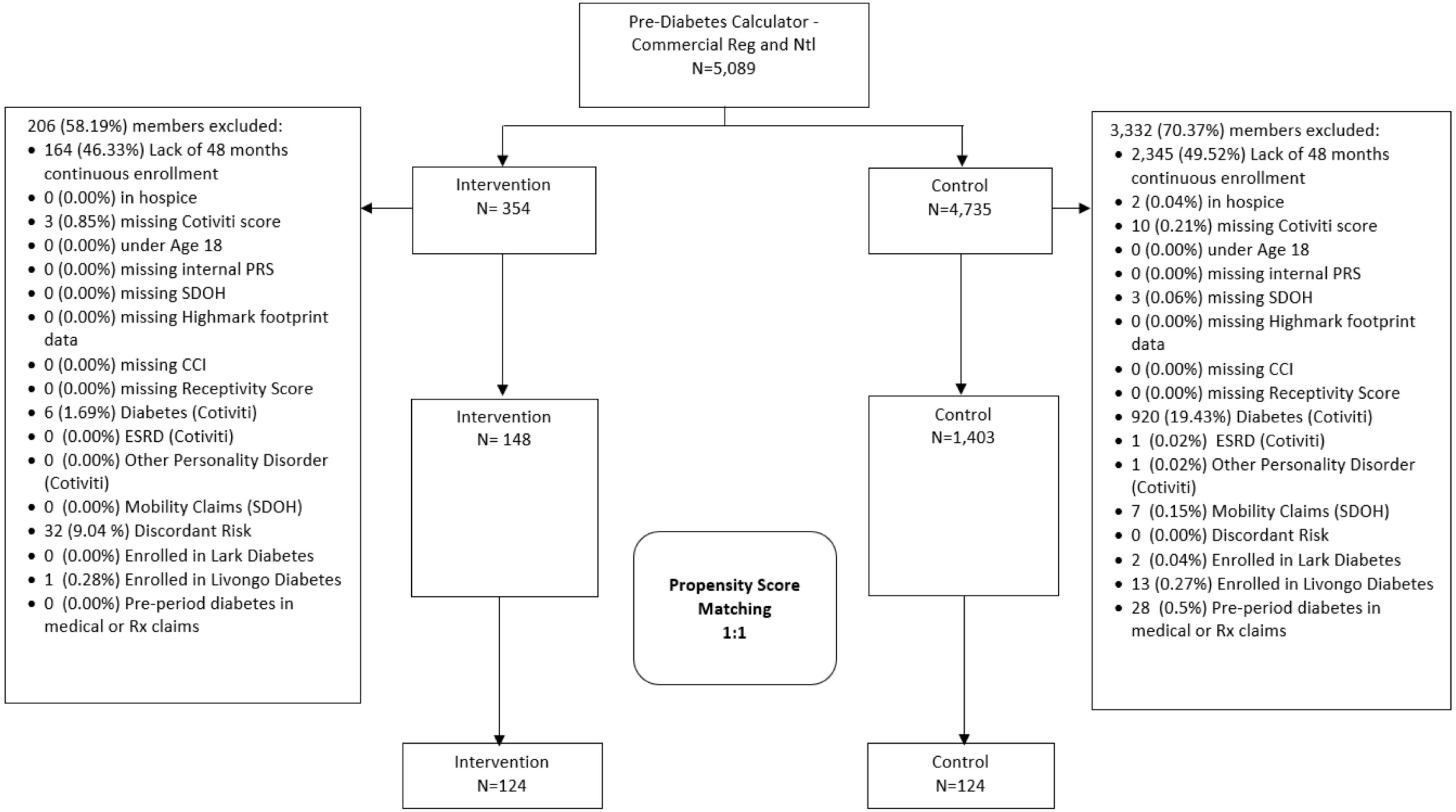

B. Medicare Advantage patients

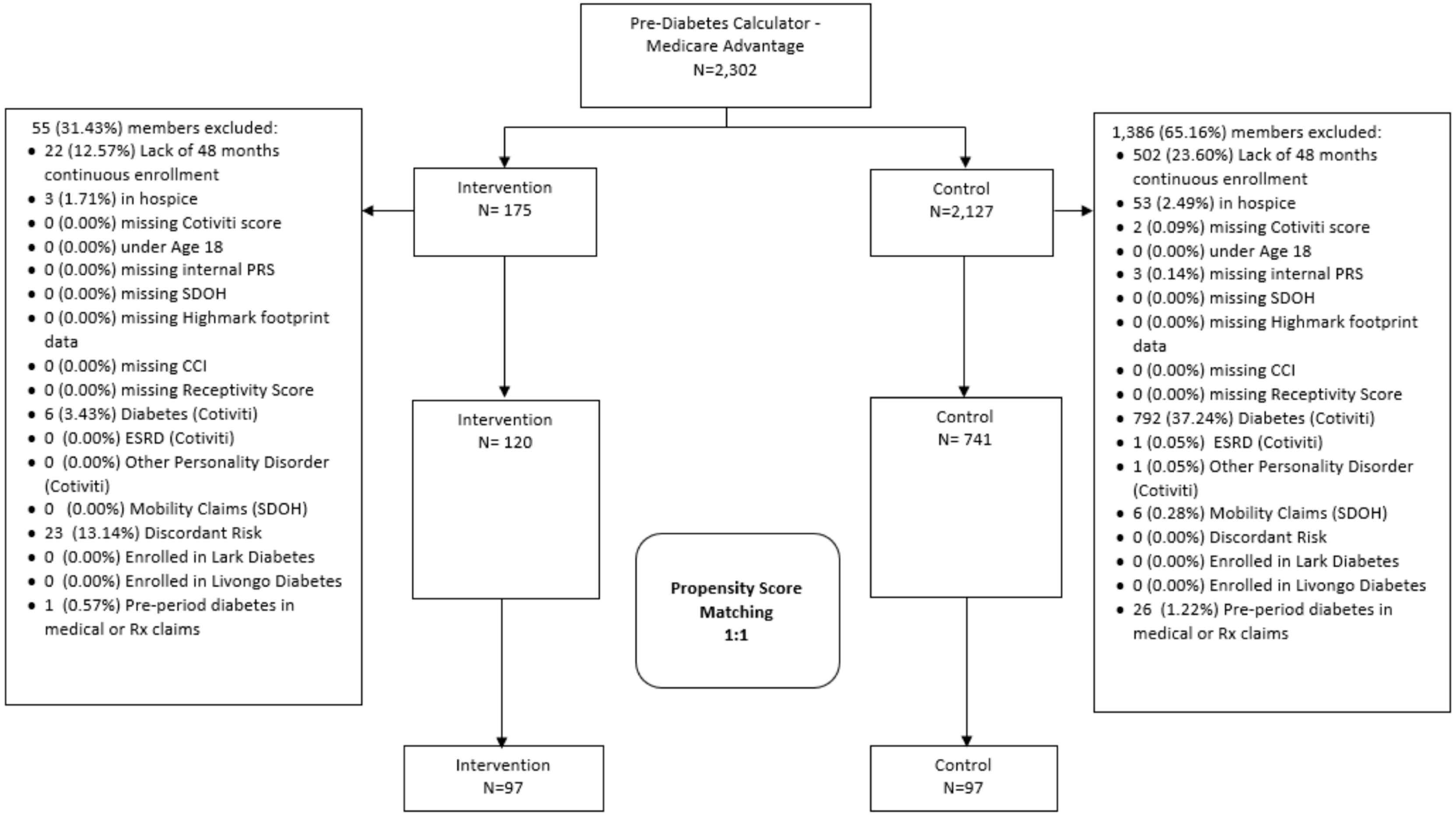

Supplement: Supplementary file 3 — File S2: lrh270087‐sup‐0003‐Supplementary_File_S2.pdf. [file LRH2-10-e70087-s002.pdf]
